# Supplementary material for: SLIMEr: probing flexibility of lipid metabolism in yeast with an improved constraint-based modeling framework
Source: BMC Syst Biol. 2019 Jan 11;13:4. doi: 10.1186/s12918-018-0673-8 (PMC6330394; doi:10.1186/s12918-018-0673-8)
Supplement: Supplementary file 1 — Supplementary material, including all supplementary tables and supplementary figures. (PDF 1090 kb) [file 12918_2018_673_MOESM1_ESM.pdf]

Supplementary material:

SLIMEr: probing flexibility of lipid metabolism in yeast with an improved constraint-based modeling framework

Benjamín J. Sánchez[1,2], Feiran Li[1,2], Eduard J. Kerkhoven[1,2] and Jens Nielsen[1,2,3(*)]

[1] Department of Biology and Biological Engineering, Chalmers University of Technology, Gothenburg, Sweden

[2] Novo Nordisk Foundation Center for Biosustainability, Chalmers University of Technology, Gothenburg, Sweden

[3] Novo Nordisk Foundation Center for Biosustainability, Technical University of Denmark, Lyngby, Denmark

(*) Corresponding author.

Correspondence: nielsenj@chalmers.se

Index

1. **Abbreviations** 3
2. **Supplementary Tables** 3
3. **Supplementary Figures** 5
4. **References** 10

List of Tables

Table S1 3

Table S2 5

List of Figures

Figure S1 5

Figure S2 6

Figure S3 7

Figure S4 8

Figure S5 9

Figure S6 9

Figure S7 10

1. Abbreviations

CL	Cardiolipin
Cer	Ceramide
DAG	Diglyceride
FVA	Flux variability analysis
GEM	Genome-scale metabolic model
IPC	Inositolphosphoceramide
LCB	Long-chain base
LCBP	Long-chain base phosphate
LPI	Lysophosphatidylinositol
M(IP)2C	Mannosyl-diinositolphosphoceramide
MIPC	Mannosyl-inositolphosphoceramide
PA	Phosphatidate
PC	Phosphatidylcholine
PE	Phosphatidylethanolamine
pFBA	Parsimonious flux balance analysis
PG	Phosphatidylglycerol
PI	Phosphatidylinositol
PS	Phosphatidylserine
SE	Ergosterol ester
TAG	Triglyceride

2. Supplementary Tables

Table S1 (next page): Molar and mass production if 1 mmol/gDWh of glucose is directed solely for each of the specific lipid species. For each lipid class, the cheapest molecule to produce in terms of mmol/gDWh and g/gDWh is highlighted in green. As it can be seen, the only case in which the highest molar production does not match the highest mass production (i.e. the cheapest molecule to produce) is for the ergosterol esters, as the backbone molecule (in this case ergosterol) is significantly larger than the other backbones present in the model. This leads to ergosteryl oleate (C18:1 molecule) to be cheaper to produce mass-wise than ergosteryl palmitoleate (C16:1) in the case of the permissive model.

Lipid	Molar production [mmol/gDWh]	Mass production [g/gDWh]
1-phosphatidyl-1D-myo-inositol (1-16:0, 2-16:1)	0.0878	0.0710
1-phosphatidyl-1D-myo-inositol (1-16:1, 2-16:1)	0.0873	0.0704
1-phosphatidyl-1D-myo-inositol (1-18:0, 2-16:1)	0.0832	0.0697
1-phosphatidyl-1D-myo-inositol (1-18:1, 2-16:1)	0.0828	0.0691
1-phosphatidyl-1D-myo-inositol (1-16:0, 2-18:1)	0.0832	0.0697
1-phosphatidyl-1D-myo-inositol (1-16:1, 2-18:1)	0.0828	0.0691
1-phosphatidyl-1D-myo-inositol (1-18:0, 2-18:1)	0.0791	0.0684
1-phosphatidyl-1D-myo-inositol (1-18:1, 2-18:1)	0.0787	0.0679
ergosteryl palmitoleate	0.0678	0.0429
ergosteryl oleate	0.0651	0.0430
palmitate	0.2125	0.0543
palmitoleate	0.2097	0.0531
stearate	0.1876	0.0532
oleate	0.1854	0.0522
phosphatidyl-L-serine (1-16:0, 2-16:1)	0.0925	0.0679
phosphatidyl-L-serine (1-16:1, 2-16:1)	0.0920	0.0673
phosphatidyl-L-serine (1-18:0, 2-16:1)	0.0875	0.0666
phosphatidyl-L-serine (1-18:1, 2-16:1)	0.0870	0.0661
phosphatidyl-L-serine (1-16:0, 2-18:1)	0.0875	0.0666
phosphatidyl-L-serine (1-16:1, 2-18:1)	0.0870	0.0661
phosphatidyl-L-serine (1-18:0, 2-18:1)	0.0829	0.0655
phosphatidyl-L-serine (1-18:1, 2-18:1)	0.0825	0.0650
phosphatidylcholine (1-16:0, 2-16:1)	0.0832	0.0609
phosphatidylcholine (1-16:1, 2-16:1)	0.0827	0.0604
phosphatidylcholine (1-18:0, 2-16:1)	0.0791	0.0588
phosphatidylcholine (1-18:1, 2-16:1)	0.0787	0.0584
phosphatidylcholine (1-16:0, 2-18:1)	0.0791	0.0601
phosphatidylcholine (1-16:1, 2-18:1)	0.0787	0.0596
phosphatidylcholine (1-18:0, 2-18:1)	0.0753	0.0582
phosphatidylcholine (1-18:1, 2-18:1)	0.0750	0.0578
phosphatidylethanolamine (1-16:0, 2-16:1)	0.0928	0.0641
phosphatidylethanolamine (1-16:1, 2-16:1)	0.0923	0.0635
phosphatidylethanolamine (1-18:0, 2-16:1)	0.0878	0.0630
phosphatidylethanolamine (1-18:1, 2-16:1)	0.0873	0.0625
phosphatidylethanolamine (1-16:0, 2-18:1)	0.0878	0.0630
phosphatidylethanolamine (1-16:1, 2-18:1)	0.0873	0.0625
phosphatidylethanolamine (1-18:0, 2-18:1)	0.0832	0.0621
phosphatidylethanolamine (1-18:1, 2-18:1)	0.0828	0.0616
triglyceride (1-16:0, 2-16:1, 3-16:0)	0.0667	0.0537
triglyceride (1-16:0, 2-18:1, 3-16:0)	0.0640	0.0534
triglyceride (1-16:1, 2-16:1, 3-16:0)	0.0664	0.0534
triglyceride (1-16:1, 2-18:1, 3-16:0)	0.0638	0.0530
triglyceride (1-18:0, 2-16:1, 3-16:0)	0.0640	0.0534
triglyceride (1-18:0, 2-18:1, 3-16:0)	0.0616	0.0530
triglyceride (1-18:1, 2-16:1, 3-16:0)	0.0638	0.0530
triglyceride (1-18:1, 2-18:1, 3-16:0)	0.0613	0.0527
triglyceride (1-16:0, 2-16:1, 3-16:1)	0.0664	0.0534
triglyceride (1-16:0, 2-18:1, 3-16:1)	0.0638	0.0530
triglyceride (1-16:1, 2-16:1, 3-16:1)	0.0661	0.0530
triglyceride (1-16:1, 2-18:1, 3-16:1)	0.0635	0.0527
triglyceride (1-18:0, 2-16:1, 3-16:1)	0.0638	0.0530
triglyceride (1-18:0, 2-18:1, 3-16:1)	0.0613	0.0527
triglyceride (1-18:1, 2-16:1, 3-16:1)	0.0635	0.0527
triglyceride (1-18:1, 2-18:1, 3-16:1)	0.0611	0.0524
triglyceride (1-16:0, 2-16:1, 3-18:0)	0.0640	0.0534
triglyceride (1-16:0, 2-18:1, 3-18:0)	0.0616	0.0530
triglyceride (1-16:1, 2-16:1, 3-18:0)	0.0638	0.0530
triglyceride (1-16:1, 2-18:1, 3-18:0)	0.0613	0.0527
triglyceride (1-18:0, 2-16:1, 3-18:0)	0.0616	0.0530
triglyceride (1-18:0, 2-18:1, 3-18:0)	0.0593	0.0527
triglyceride (1-18:1, 2-16:1, 3-18:0)	0.0613	0.0527
triglyceride (1-18:1, 2-18:1, 3-18:0)	0.0591	0.0524
triglyceride (1-16:0, 2-16:1, 3-18:1)	0.0638	0.0530
triglyceride (1-16:0, 2-18:1, 3-18:1)	0.0613	0.0527
triglyceride (1-16:1, 2-16:1, 3-18:1)	0.0635	0.0527
triglyceride (1-16:1, 2-18:1, 3-18:1)	0.0611	0.0524
triglyceride (1-18:0, 2-16:1, 3-18:1)	0.0613	0.0527
triglyceride (1-18:0, 2-18:1, 3-18:1)	0.0591	0.0524
triglyceride (1-18:1, 2-16:1, 3-18:1)	0.0611	0.0524
triglyceride (1-18:1, 2-18:1, 3-18:1)	0.0589	0.0521

Table S2: Average absolute error [mg/gDW] in predicting the experimental lipid profile.

Condition	WT 24ºC	WT 37ºC	Δ*elo1* 24ºC	Δ*elo1* 37ºC	Δ*elo2* 24ºC	Δ*elo2* 37ºC	Δ*elo3* 24ºC	Δ*elo3* 37ºC
**Permissive GEM**	0.85	0.82	0.89	0.81	0.94	0.89	0.71	0.74
**Enhanced GEM**	0.54	0.51	0.60	0.51	0.65	0.59	0.53	0.58

3. Supplementary Figures

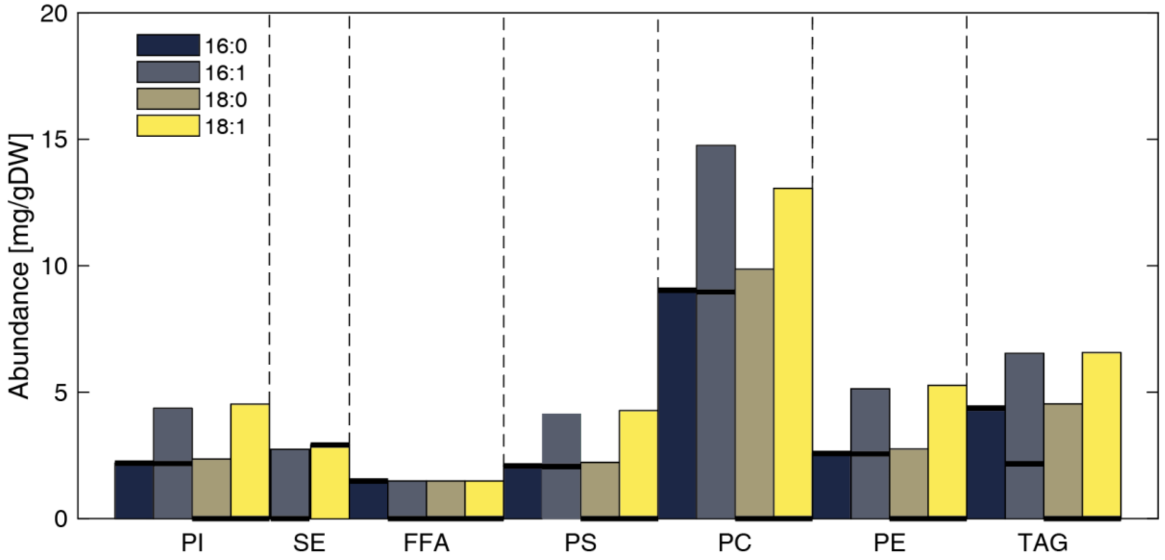

Figure S1: Breakdown of the acyl chain distribution and variability predicted by the **permissive GEM**, for each experimentally detected lipid class. Thick dark lines correspond to pFBA predictions, while the FVA allowed ranges are shown with colored bars.

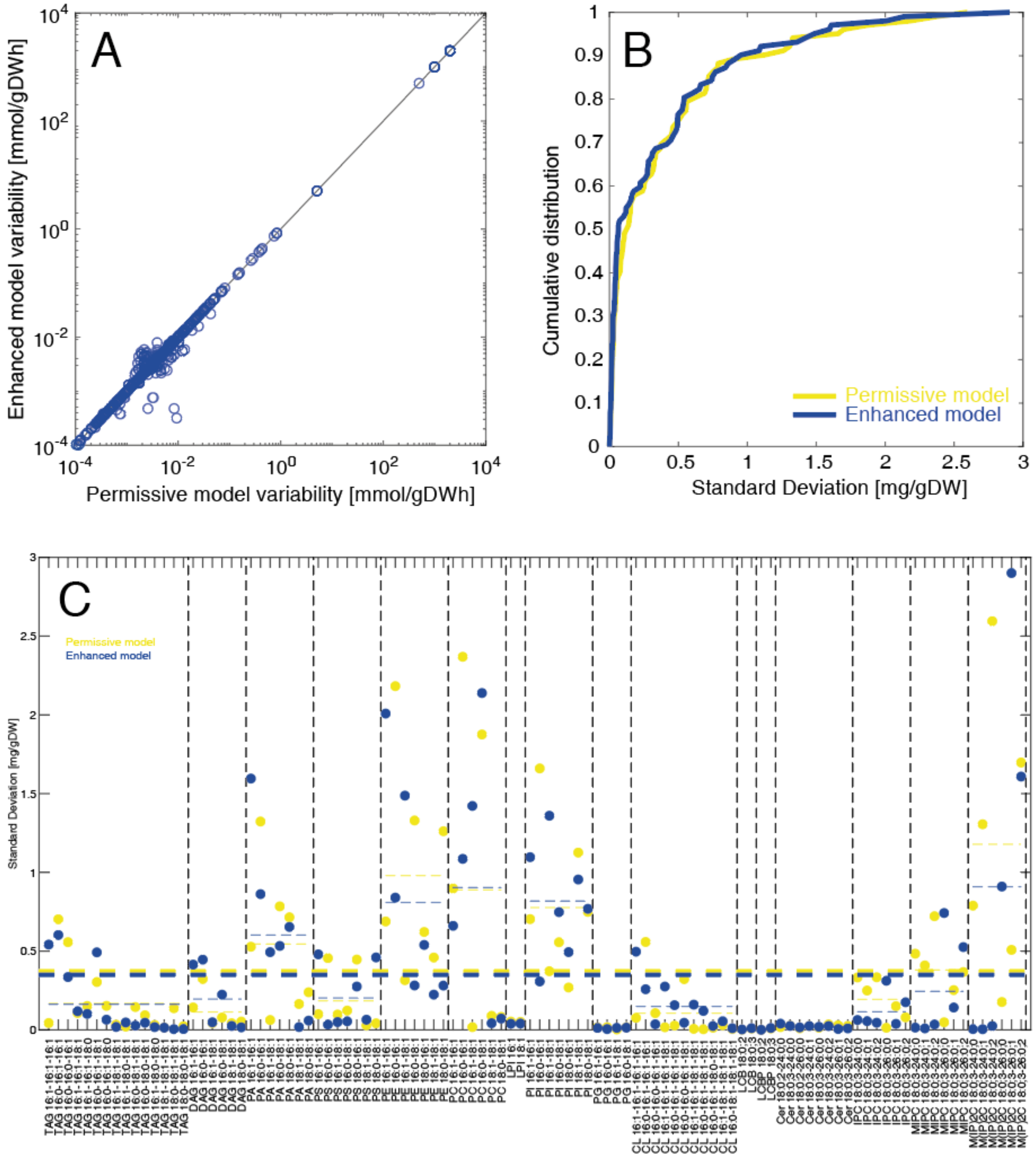

Figure S2: The variability of simulations between the enhanced and permissive models are similar. **(A)** Comparison of the variability span (i.e. maximum – minimum value) for all flux predictions under reference conditions (Lahtvee *et al*, 2017). **(B)** Cumulative distributions of the variability from the random sampling simulations under reference conditions (Ejsing *et al*, 2009). **(C)** Breakdown by lipid species of the variability from the random sampling simulations in (B). Thin colored segmented lines indicate medians by lipid class, and the thick ones show the overall median across all lipids.

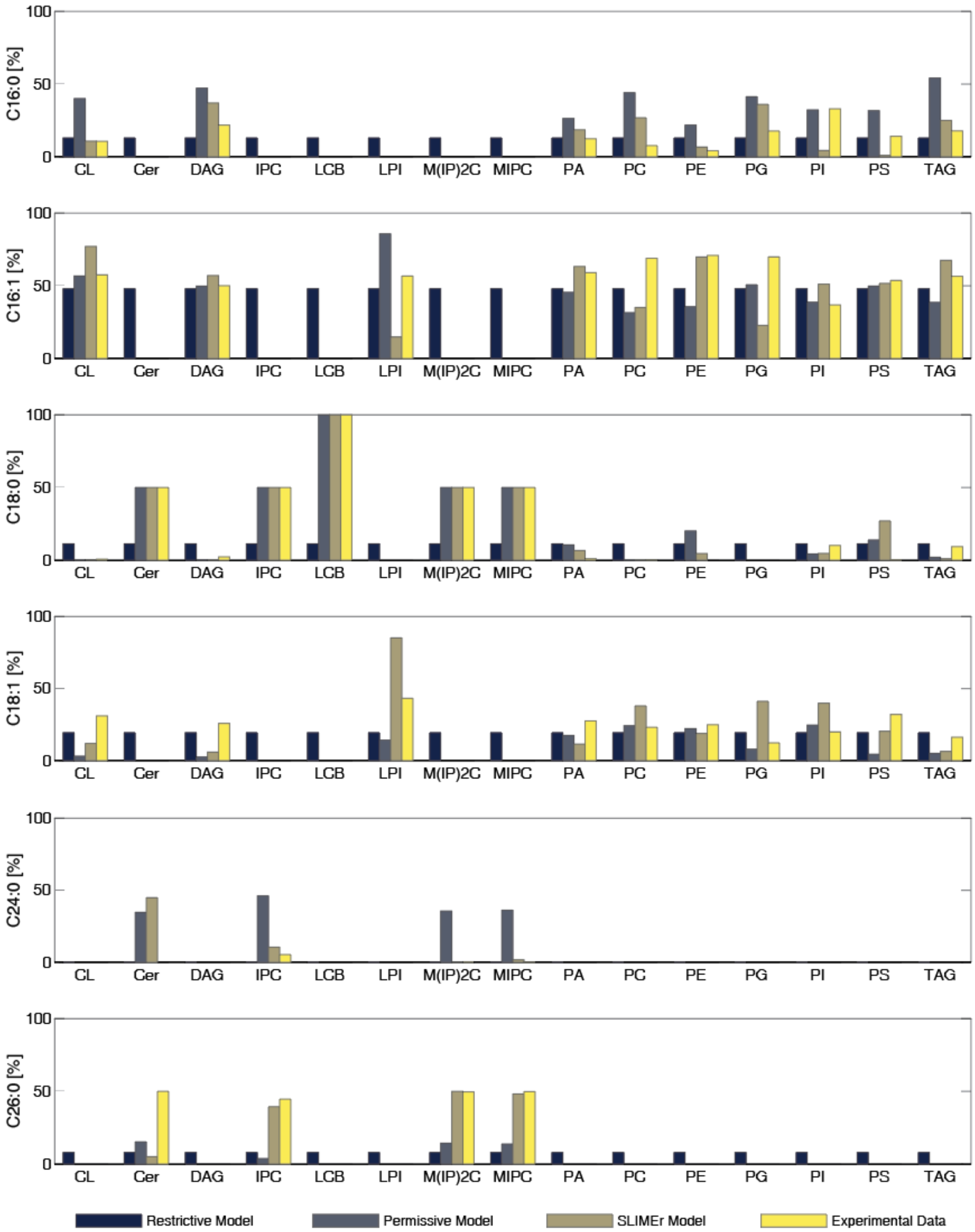

Figure S3: Molar proportions of each acyl chain in each lipid class, for (i) a hypothetical model built with the restrictive approach, i.e. every lipid class has the same acyl chain fractions based on the average from the experimental data; (ii) the permissive model; (iii) the enhanced model with SLIME reactions, and (iv) the experimental data.

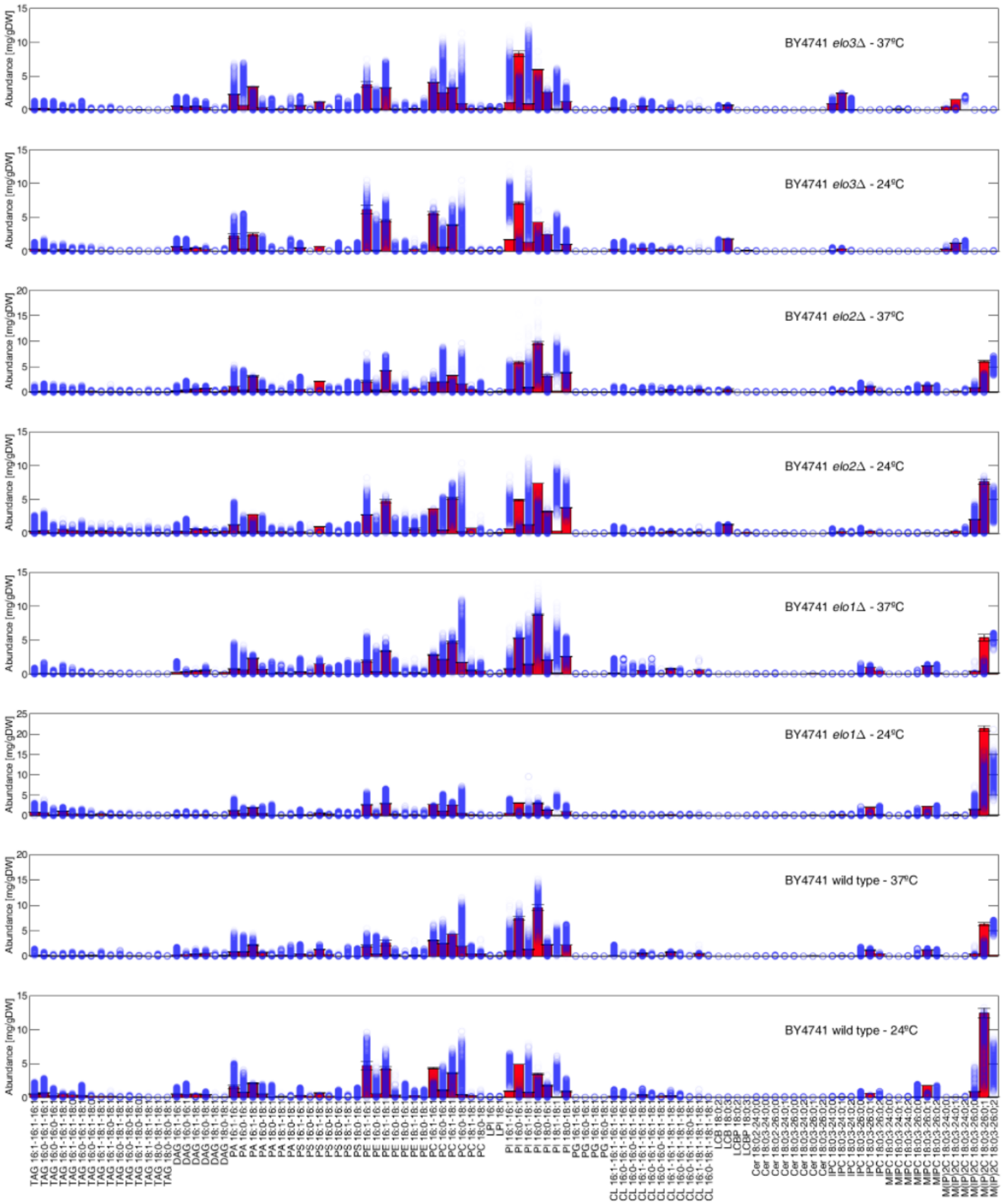

Figure S4: The lipid composition of 10,000 simulations of the enhanced model achieved with random sampling are presented for each specific lipid species (blue circles), compared to the actual measured experimental values (red bars) (Ejsing *et al*, 2009), for 8 different conditions (1 wild type + 3 knockouts under 2 different temperatures).

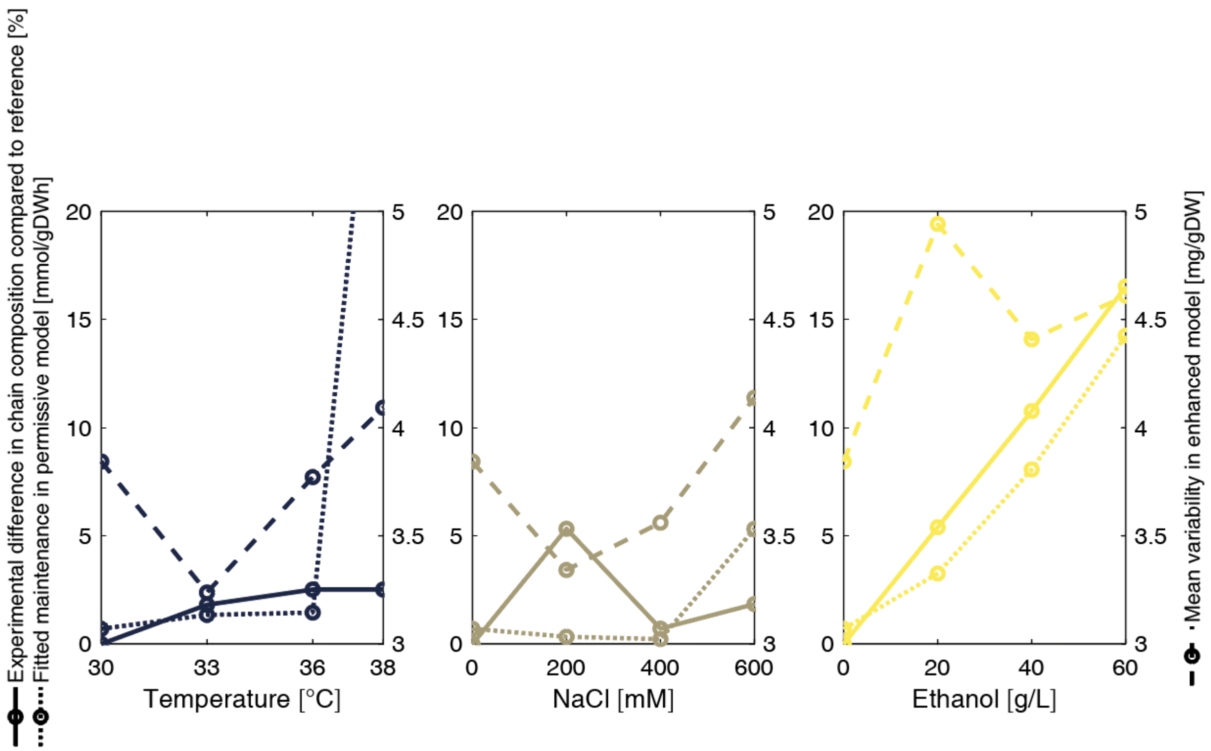

Figure S5: Experimental difference in chain composition compared to reference conditions (continuous lines) (Lahtvee *et al*, 2017), fitted non-growth associated maintenance in permissive model (dotted lines) and mean lipid variability in the enhanced model (segmented lines), for all 3 types of stresses.

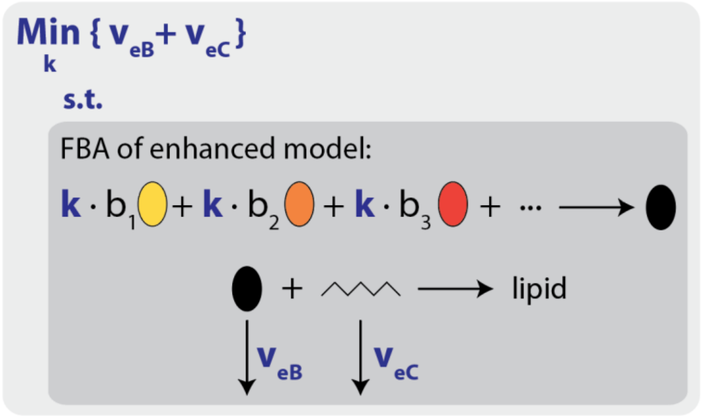

Figure S6: Optimization problem solved for finding the scaling factor for the experimental data. Here it is shown for scaling the lipid classes; for the acyl chain data it would be the same but rescaling the other pseudo-reaction.

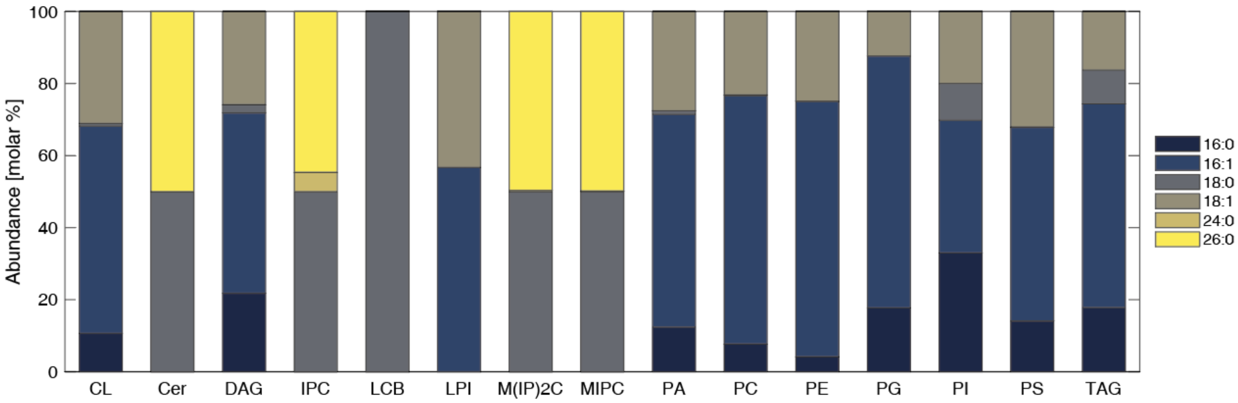

Figure S7: Experimental acyl chain distribution across all lipid classes of yeast, measured under wild type growth (Ejsing *et al*, 2009).

4. References

Ejsing CS, Sampaio JL, Surendranath V, Duchoslav E, Ekroos K, Klemm RW, Simons K & Shevchenko A (2009) Global analysis of the yeast lipidome by quantitative shotgun mass spectrometry. *Proc. Natl. Acad. Sci.* **106:** 2136–2141

Lahtvee PJ, Sánchez BJ, Smialowska A, Kasvandik S, Elsemman IE, Gatto F & Nielsen J (2017) Absolute Quantification of Protein and mRNA Abundances Demonstrate Variability in Gene-Specific Translation Efficiency in Yeast. *Cell Syst.* **4:** 495–504.e5
